# Heart Failure and PAHs, OHPAHs, and Trace Elements Levels in Human Serum: Results from a Preliminary Pilot Study in Greek Population and the Possible Impact of Air Pollution

**DOI:** 10.3390/molecules26113207

**Published:** 2021-05-27

**Authors:** Eirini Chrysochou, Panagiotis Georgios Kanellopoulos, Konstantinos G. Koukoulakis, Aikaterini Sakellari, Sotirios Karavoltsos, Minas Minaidis, Evangelos Bakeas

**Affiliations:** 1 Laboratory of Analytical Chemistry, Department of Chemistry, National and Kapodistrian University of Athens, Panepistimiopolis, 15784 Athens, Greece; eirinichr@chem.uoa.gr (E.C.); gpkan@chem.uoa.gr (P.G.K.); kkoukoulakis@chem.uoa.gr (K.G.K.); 2 Laboratory of Environmental Chemistry, Department of Chemistry, National and Kapodistrian University of Athens, Panepistimiopolis, 15784 Athens, Greece; esakel@chem.uoa.gr (A.S.); skarav@chem.uoa.gr (S.K.); 3 General Hospital “LAIKO”, 11527 Athens, Greece; minaidis64@hotmail.com

**Keywords:** cardiovascular diseases (CVDs), heart failure, polycyclic aromatic hydrocarbons (PAHs), trace elements, serum

## Abstract

Cardiovascular diseases (CVDs) have been associated with environmental pollutants. The scope of this study is to assess any potential relation of polycyclic aromatic hydrocarbons (PAHs), their hydroxylated derivatives, and trace elements with heart failure via their direct determination in human serum of Greek citizens residing in different areas. Therefore, we analyzed 131 samples including cases (heart failure patients) and controls (healthy donors), and the respective demographic data were collected. Significantly higher concentrations (*p* < 0.05) were observed in cases’ serum regarding most of the examined PAHs and their derivatives with phenanthrene, fluorene, and fluoranthene being the most abundant (median of >50 μg L^−1^). Among the examined trace elements, As, Cd, Cu, Hg, Ni, and Pb were measured at statistically higher concentrations (*p* < 0.05) in cases’ samples, with only Cr being significantly higher in controls. The potential impact of environmental factors such as smoking and area of residence has been evaluated. Specific PAHs and trace elements could be possibly related with heart failure development. Atmospheric degradation and smoking habit appeared to have a significant impact on the analytes’ serum concentrations. PCA–logistic regression analysis could possibly reveal common mechanisms among the analytes enhancing the hypothesis that they may pose a significant risk for CVD development.

## 1. Introduction

Air pollution is a major public health problem with a plethora of consequences on humans and other living beings [1]. An estimated measure of more than 4.2 million annual global premature deaths related to air pollution has been reported [2]. Outdoor air pollution is considered the fifth greatest risk factor for all-cause mortality, which is higher than the acknowledged risk factors, including poor diet and low exercise, and the first among environmental risk factors [3]. Air pollution consists of plenty of diverse pollutants partitioned in the gas phase, such as volatile organic compounds (VOCs), nitrogen oxides (NO_x_), sulfur dioxide (SO_2_), carbon monoxide (CO), etc. [4,5,6] and in the particle phase including polycyclic aromatic hydrocarbons (PAHs), trace elements, polychlorinated biphenyls (PCBs), etc. [7,8]. As a result of the presence of different harmful substances, the International Agency for Research on Cancer (IARC) has classified air pollution as a human carcinogen [9]. Globally, over 90% of individuals live in areas where air pollution levels exceed the World Health Organization (WHO) guidelines [2]. The well-known “Harvard Six Cities Study” was the origin of the link of air pollution to mortality from lung cancer and cardiopulmonary disease [10]. In this light, many studies addressed geographic differences of exposures, for example PAHs and particulates exposures in urban and rural areas of Czech Republic [11], or temporal sources of pollution, such as the New York World Trade Center disaster, which was a transient event of PAHs, dioxins, and inorganic dusts exposure of a well-defined population [12], with air pollution being associated with mortality [13,14,15,16]

Although the lung is the main receptor of air contaminants, air pollution is significantly associated with cardiovascular diseases (CVDs) [17,18]. In particular, exposure to atmospheric particulate matter (PM) has been correlated with increased arrhythmia incidents, carotid intima-media thickness, which is a marker of subclinical atherosclerosis, with the progression of inflammation and hypertension [19,20,21], as well as with reduced heart rate variability (HRV) [22] with particles with diameter <0.3 μm being the most crucial PM fraction to the reduction of the cardiac autonomic function [23]. Moreover, a 10–30% increase of the death risk from ischemic heart disease per 10 μg m^−3^ increase of PM_2.5_ (particles with aerodynamic diameter <2.5 μm) has been estimated [24]. Generally, air pollution’s implications on CVDs may lead to higher mortality rates than those caused by the air pollution impact on respiratory diseases [3]. In Europe, about 3.9 million deaths annually have been attributed to CVDs making them the leading cause of mortality, with more than 85 million European citizens living with CVDs during 2015 [25].

PM-bound PAHs are associated with CVDs, with studies involving humans and other mammals underlining the link of tricyclic PAHs, and especially phenanthrene with arrhythmias, the aggravation of heart failure, heart attacks, and other complications involving atherosclerosis and ischemia [17,26]. Occupational exposure to PAHs has been also linked with alterations in cardiac autonomic function, as implied by the decreased HRV [27]. The cardiovascular toxicity of PAHs involves aryl hydrocarbon receptor (AhR), reactive oxygen species (ROS), and/or reactive electrophilic metabolites, with the cardiotoxic effects not being limited to benzo[a]pyrene (BaP) but also to other PAHs including pyrene (PYR), phenanthrene (PHE), and benzo[e]pyrene (BeP) [28]. PAHs mixtures and especially PHE cause cardiotoxicity independent of the (AhR) pathway with various toxicant and cellular pathways involving atherosclerosis, cardiac arrhythmias, and cardiac hypertrophy [26]. For instance, the DNA methylation, caused by exposure to PHE, may induce cardiac hypertrophy with a mechanism that involves the reduction of the miR-133a expression [29]. PAHs constitute a group of compounds formed by the incomplete combustion of carbonaceous material, which can be emitted into the atmosphere from both natural and anthropogenic sources, including vehicular emissions, domestic heating, power plants, tobacco smoke, and solid waste incineration [30,31]. In developing countries, organic wastes burning for domestic needs such as cooking [32] is another source of PAHs. After exposure, PAHs bound to the smallest sized particles in PM_2.5_ can enter the systemic circulation un-metabolized and reach various organs [26].

Apart from PAHs, environmental trace elements are a noteworthy but overlooked source of CVDs risk [33]. Various studies implied that trace elements including As, Cd, Hg, and Pb may constitute an important factor to CVDs development [34,35,36,37]. Although other trace elements such as Co, Cu, Fe, Se and Zn are essential for the human organism [38,39,40,41], high exposure to them is also associated with the risk of CVD development [24]. Airborne particle-bound trace elements have both natural and anthropogenic origin, including natural dust emissions [42], coal [43] and oil combustion [44], the production of iron, steel, cast iron, etc. [45]. However, metal exposure is usually neglected by the agencies that produce guidelines about cardiovascular prevention [33].

There is adequate data based on measurements of urinary metabolites of PAHs trying to link PAHs levels with CVDs [46,47,48] and other diseases, including rheumatoid arthritis [49] and diabetes [50]. However, data of PAHs levels in plasma and serum related to CVDs are limited [26]. The determination of PAHs in different human matrices provides different information based on the selected matrix. For example, urine samples are more closely related with metabolites derived from biotransformation procedures; hair samples are considered ideal for long-term exposure studies; whereas blood, serum, saliva, or exhaled breath samples are more associated with unmetabolized PAHs recent exposure [51]. Moreover, blood-borne PAHs compared to their respective metabolites or adducts are less susceptible to variability from inter-subject differences in metabolism and excretion [52]. In addition, serum is a more familiar and efficient way for the monitoring of PAHs compared to other tissues [53]. PAHs have been measured in serum samples not only in an effort to investigate any potential association with different types of cancer such as leukemia [54] and bladder cancer [55] but also for the estimation of background burden values [56,57]. Moreover, PAHs have been measured at the maternal serum in order to investigate the possible transplacental transfer from the mother to the fetus [58], while others have proposed an inversed trend of maternal serum PAHs and a decreased birth weight [59].

Trace elements are measured in various biological samples, including blood, serum, erythrocytes, and urine, with the whole blood being the preferred matrix used for the biomonitoring of the toxic Pb, Cd, and Hg as they are concentrated in the erythrocytes. The determination of serum trace elements is also prone to errors due to hemolysis, which may lead to possible errors in the results [60]. However, the elemental composition of serum is widely studied, as it provides important insights for the state of a human organism [61] and because it is the most exchangeable blood compartment [60]. Serum trace elements’ levels have been associated with various CVDs, including coronary artery disease (CAD) [62], carotid artery atherosclerosis in maintenance hemodialysis patients [63], and other diseases, such as asthma, various allergic diseases [64], and different types of leukemia [65,66].

Heart failure is a clinical syndrome provoked by multiple causes [67], with the CAD and arterial hypertension being the leading causes and with heart dysfunction, valvular disease, tachyarrhythmias, diabetes mellitus, myocarditis, and infiltrative disorders being some of the other causes [68].

In this perspective, the aim of this work is the determination and comparison of PAHs’ and OHPAHs’ trace elements’ concentrations in human serum of heart failure patients and healthy donors, all residing in different areas of Greece, and the investigation of the potential impact of different environmental factors including smoking habit and area of residence.

## 2. Results and Discussion

### 2.1. Cases and Controls

**Figure 1:**
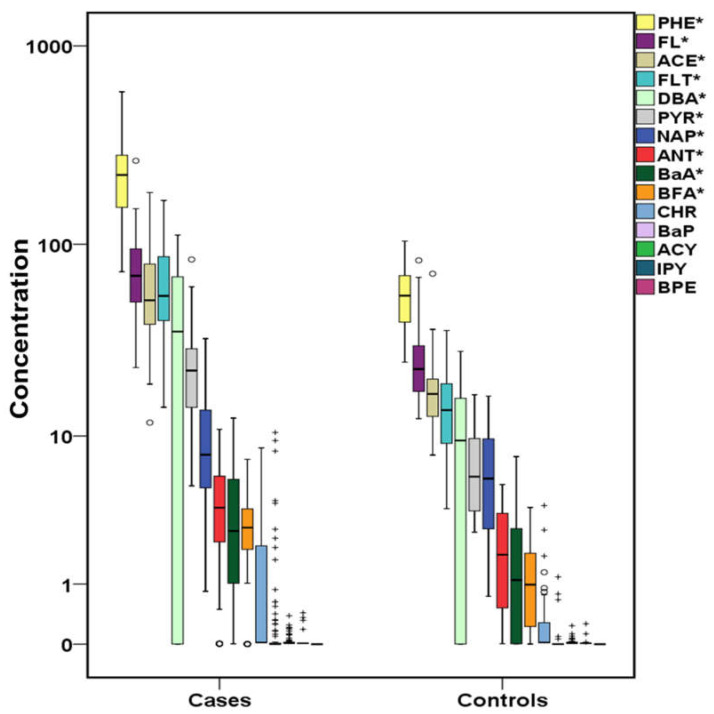
Variation of PAHs (μg L^−1^) both for cases and controls (^+^ outliers, ^o^ values above 3rd quartile and * *p*-value < 0.05) in logarithmic scale (PHE:phenanthrene, FL:fluorene, ACE:acenaphthene, FLT:fluoranthene, DBA:dibenzo[a,h]anthracene, PYR:pyrene, NAP:naphthalene, ANT:anthracene, BaA:benzo[a]anthracene, BFA:benzo[b,k]fluoranthenes, CHR:chrysene, BaP:benzo[a]pyrene, ACY:acenaphthylene, IPY:indeno[1,2,3 cd]pyrene, BPE:benzo[ghi]perylene).

**Figure 2:**
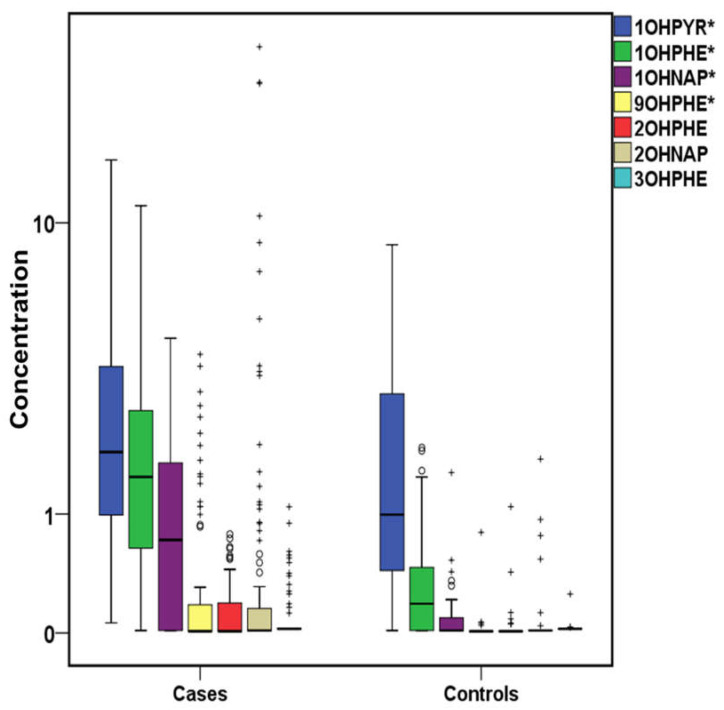
Variation of OHPAHs (μg L^−1^) both for cases and controls (^+^ outliers, ^o^ values above 3rd quartile and * *p*-value < 0.05) in logarithmic scale (1OHPYR:1-hydroxypyrene, 1OHPHE:1-hydroxyphenanthrene, 1OHNAP:1-naphthol, 9OHPHE:9-hydroxyphenanthrene, 2OHPHE:2-hydroxyphenanthrene, 2OHNAP:2-naphthol, 3OHPHE:3–hydroxyl-phenanthrene).

**Figure 3:**
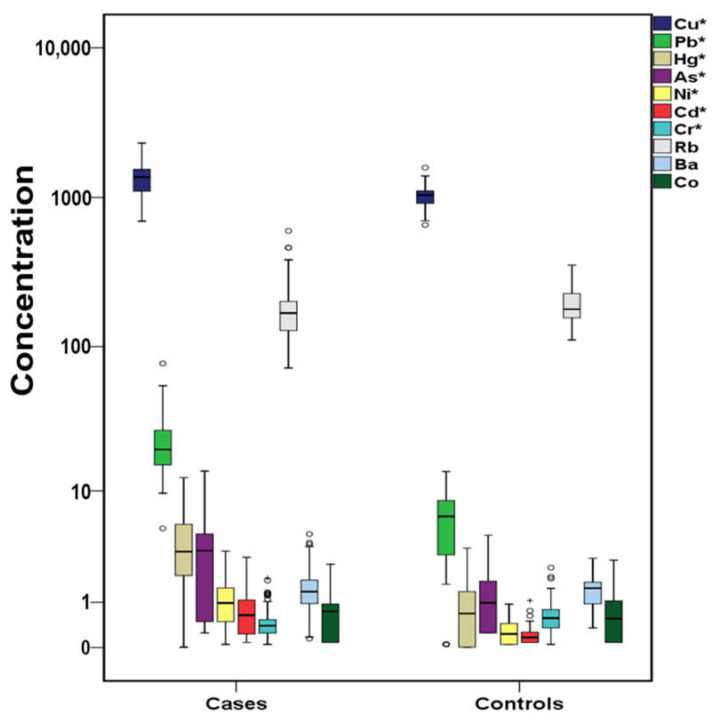
Variation of trace elements (μg L^–1^) both for cases and controls (^+^ outliers, ^o^ values above 3rd quartile and * *p*-value < 0.05) in logarithmic scale.

The total concentrations of PAHs, OHPAHs, and trace elements for cases and controls are presented in Figure 1, Figure 2 and Figure 3. More detailed information about the mean, median, and concentration ranges of the analytes is shown in Tables S1–S3.

#### 2.1.1. PAHs

**Figure 4:**
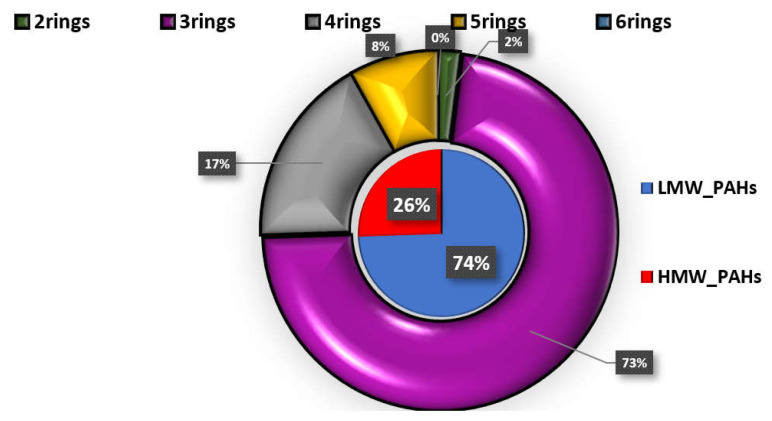
Distribution of HPAHs and LPAHs in cases’ serum.

Almost the 70% of the studied PAHs presented detection frequencies over 65% (Table S1). In particular, naphthalene (NAP), acenaphthene (ACE), fluorene (FL), PHE, fluoranthene (FLT) and PYR were detected in 100% of both cases’ and controls’ samples. Anthracene (ANT), benzo[a]anthracene (BaA), benzo[b,k]fluoranthenes (BFA), and dibenzo[a,h]anthracene (DBA) were found at detection rates varying from 65.7% (DBA-control samples) to 93.7% (BFA-cases samples). Lower detection rates were found for chrysene (CHR) (35.4% in cases and 28.6 in controls), while the rest of the studied PAHs, including BaP, acenaphthylene (ACY), indeno[1,2,3 cd]pyrene (IPY), and benzo[ghi]perylene (BPE) were detected at ≤25% of the samples. As shown in Figure 1, PHE was the dominant PAH in both cases’ and controls’ serum followed by FL, with the concentrations in cases’ serum being approximately four and three times higher, respectively (*p* < 0.05) (Table S1). Significantly higher concentrations in cases’ samples were also observed for the following PAHs with a concentration descending sequence of ACE, FLT, DBA, PYR, NAP, ANT, BaA, and BFA with FLT, PYR, and DBA median values being almost 4-fold higher than those observed in controls’ serum. CHR, BaP, ACY, and IPY did not differ significantly (*p* > 0.05), since in many cases and controls samples, their concentration was below the limits of detection (LoD). The relative profile of PAHs, regarding the number of rings in each molecule, in cases’ serum is displayed in Figure 4. The dominance of 3-ring-PAHs is highlighted as they accounted for the 73% of the total measured PAHs. As a result, the contribution of low molecular weight PAHs (LMW PAHs) was almost three times higher than that of high molecular weight PAHs (HMW PAHs). Consequently, the significantly higher concentrations of the majority of PAHs and especially LMW PAHS, detected in this work, may play a pivotal role in the development of heart failure.

High PAHs detection frequencies combined with higher percentages of LMW compared to HMW PAHs in serum have been reported in many other studies [54,69,70]. However, relatively low rates (<20%) have been also reported in a pilot study in a rural area of Egypt [71]. PAHs have been found in significantly higher concentrations in the serum of patients than those of corresponding controls’ serum in the cases of bladder cancer, leukemia, and polycystic ovary syndrome [54,55,72].

#### 2.1.2. OHPAHs

Urinary OHPAHs are considered as PAH metabolites, and as a result, most studies that related PAHs exposure and CVDs have studied OHPAHs in urine [46,47,73,74,75]. However, concentrations of OHPAHs and/or the parent ones in urine do not only depend on the external exposure but also on difference in the metabolism and bioconversion procedures in each individual organism [76]. Therefore, noteworthy results can also be extracted from serum determination, too [54,72]. The detection frequencies of OHPAHs are shown in Table S2. In cases’ serum, OHPAHs were detected at higher rates than those measured in controls’, with 1-hydroxypyrene (1OHPYR) being found at all samples followed by 1-hydroxyphenanthrene (1OHPHE) and 1-napthol (1OHNAP) with respectively equal rates of 96.9 and 65.6%. The rest of the studied OHPAHs such as 9-hydroxyphenanthrene (9OHPHE), 2-hydroxyphenanthrene (2OHPHE), 2-naphthol (2OHNAP), and 3-hydroxyphenanthrene (3OHPHE) were detected at <37% of the samples. However, in controls’ serum, the detection frequencies ranged from 8.57% (3OHPHE) to 77.1% (1OHPYR). Higher detection frequencies of serum OHPAHs have been found in other studies, from 70% (9OHPHE) to 100% (2OHNAP) in cases’ samples and from 50% (9OHPHE) to 100% (2OHNAP) in that of controls [72]. Nevertheless, lower rates were found in our previous study (40–70% in cases and <25% in controls) [54]. In general, OHPAHs presented lower concentrations than the parent ones. Significantly (*p* < 0.05) higher concentrations in cases’ serum were observed, in descending order, for 1OHPYR, 1OHPHE, 1OHNAP, 9OHPHE, as well as ΣOHPAHs (Table S2, Figure 2). 1OHPYR was the most abundant with a median of 1.87 μg L^−1^ followed by 1OHPHE and 1OHNAP with median values 1.48 and 0.71 μg L^−1^, respectively. 2OHNAP, 2OHPHE, and 3OHPHE did not differ significantly, as in many samples, their values were found below the LoD.

Although data concerning OHPAHs serum levels are sparse, some of them appear to be significantly higher in the serum of acute leukemia patients and women with polycystic ovary syndrome than that of the respective control subjects [54,72].

#### 2.1.3. Trace Elements

Trace elements were widely detected in the serum samples, with detection frequencies over 73.9% in cases’ serum and from 57.1% in control’s serum (Table S3). Significantly higher (*p* < 0.05) concentrations in cases’ serum were observed for copper (Cu), lead (Pb), mercury (Hg), arsenic (As), nickel (Ni), and cadmium (Cd), while higher, but not significantly (*p* > 0.05), concentrations were found for cobalt (Co). On the contrary, chromium (Cr), rubidium (Rb), and barium (Ba) were found at higher levels in controls’ serum; the difference was statistically significant (*p* > 0.05) only in the case of Cr though (Figure 3, Table S3). Recent reviews have highlighted the association of both known toxic and essential metals with CVDs development [77,78]; thus, the elements that were observed with significantly different concentrations in cases’ or control’s serum will be separately discussed.

Arsenic (As) is a toxic metalloid that enters the organisms through food, drinking water, cigarette smoke, and through inhalation of particle bound As [7,36,79,80]. According to the U.S Agency for Toxic Substances and Disease Registry, the safe level for As in human blood is suggested to be less than 70 μg L^−1^ [81]. The As levels presented in this work are greatly lower than the suggested value; however, As concentrations in cases’ serum were approximately 3.5 times higher than those measured in controls (median of 3.39 versus 0.98 μg L^−1^). In other studies, As was measured in the serum of 17–20-year-old students and was found at lower levels with median concentrations from 2.43 to 2.81 μg L^−1^, with the highest As values being attributed to seafood intake [79]. Relatively higher concentrations of As were measured in whole blood samples of kids with learning disorders (mean of 12.1 ± 5.19 μg L^−1^) which were significantly higher than the corresponding control values (9.73 ± 4.39 μg L^−1^) [82]. Moreover, significantly higher concentrations of As were found in the serum of chronic kidney disease patients receiving continuous ambulatory peritoneal dialysis (mean of 3.79 μg L^–1^) than those found in the respective control samples (0.52 μg L^–1^), which was related with the increased CVD risk of the specific target group [83]. 

Regarding the toxic Cd, WHO has evaluated the values of 0.1 μg to 4 μg L^–1^ of the Cd blood concentrations characterizing a healthy and unexposed adult [84]. Although our findings are under the limits, the observed statistical difference between cases (median of 0.64 μg L^–1^) and controls (median of 0.17 μg L^–1^) can not be ruled out. In other studies, the Cd serum levels of CAD patients were found to be higher than those of the negative subjects with a mean of 2.44 μg L^–1^ versus 1.15, although the difference was not significant [62]. However, the Cd serum levels of patients on maintenance hemodialysis (HD) were significantly higher than those of the control group, which was independently associated with carotid atherosclerosis: a disease common in the specific patient group [69]. Additionally, Cd exposure was associated with atherosclerosis cardiovascular disease (ASCVD) through the strong relationship of Cd blood levels from approximately 2500 individuals with the 10-year ASCVD risk scores, using risk prediction models [85].

Hg is an easily accessible toxic metal with various intake pathways, such as air, water, food, vaccines, pharmaceuticals, and cosmetics, with its cardiotoxic effects being strongly associated with hypertension, coronary heart disease, myocardial infarction, cardiac arrhythmias, carotid artery obstruction, cerebrovascular accident, and generalized atherosclerosis [86]. The recommended, by the U.S. National Academy of Sciences (NAS), average level of Hg in human blood is below 5 μg L^–1^ [87]. In the current work, 29 out of 96 cases’ samples, i.e., 30.2%, presented concentrations above the proposed level, with the median concentration, however, being below the limit (3.33 μg L^–1^). Significantly lower concentrations (*p* < 0.05) were measured in control samples varying from below the detection limits to 3.57 μg L^–1^, with a median value of 0.46 μg L^–1^. Elevated concentrations were also found in the serum of CAD patients (8.19 μg L^–1^), which were significantly higher than the respective concentrations of controls’ serum (4.11 μg L^–1^) [62]. Similar to Cd, Hg blood levels were also linked with the ASCVD risk in the Korean population [85]. Relatively high concentrations of blood Hg (mean of 102 ± 55.8 μg L^–1^) have been reported for workers in Artisanal and Small-Scale Gold Mining (ASGM) operations in Ghana, where Hg exposure was notably elevated [88]. Although the cardiovascular implications of Hg have been evaluated [89] its biokinetics and actions in the CV system are still quite elusive [90].

Regarding Pb, there is sufficient evidence for adverse health effects in children and adults at blood levels <50 μg L^–1^ [91]. Pb in the serum samples varied from 5.18 to 77.0 μg L^–1^, with a median of 19.8 μg L^–1^ which is significantly higher (*p* < 0.05) than those found for healthy donors’ samples (median of 6.44 μg L^–1^). In other studies, Pb has been also found in the serum of CAD patients with a mean of 8.19 μg L^–1^, which is over two times higher than the respective control samples (mean of 3.69 μg L^–1^) [62]. Pb competes with the essential metals, such as calcium (Ca), iron (Fe), and zinc (Zn), as it is able to bind and/or interact, with parallel ways as the latter, with the same enzymes resulting in the inhibiting of the enzyme’s ability to catalyze its normal reactions [92]. The general mechanisms to CVD development, among others, include the induction of oxidative stress, impairment of the nitric oxide system, the increased generation of ROS, changes in the Ca^+2^ transport and intracellular distribution, etc. [93,94]. Pb blood levels with a geometric mean of 20.7 μg L^–1^ have been associated with the prevalence of peripheral arterial disease in the US population [95] and with the ASCVD risk in the Korean population [85]. Pb along with Cd have been also found in significantly higher concentration in the serum of acute hemorrhagic stroke patients than the corresponding controls’ serum, indicating a possible association with this stroke subtype [96].

Apart from the known toxic metals, imbalanced levels of essential metals such as Cr, Co, Cu, magnesium (Mg), Ni, selenium (Se), tungsten (W), and Zn are significantly associated with CVDs [78]. As a result, the significantly higher concentrations (*p* < 0.05) observed, in this study, for Cu and Ni might have a role in heart failure development. Significantly higher concentrations of Co and Cu were also noticed in the serum of HD patients than those of controls’ samples having a plausible association of increased CVD risk [69]. Elevated Cu levels in serum (median of 1175 μg L^–1^) were found to be associated with CVDs among US adults [97]. In another study of 4035 middle-aged men, increased serum Cu levels were linked with a 30% increase in CV mortality [98], while other papers underline the relation of circulating blood levels of the essential Ni and Cr with atherosclerotic plagues in elderly [99]. However, as far as Cr is concerned, lower concentrations have been measured in the serum of CVD patients than those of the healthy controls [100], which is in agreement with the findings of this study. Particularly, Cr median values were 0.40 μg L^–1^ in cases’ serum versus 0.57 μg mL^–1^ in controls (*p* < 0.05). In other works, blood and scalp hair Cr was found at lower levels of myocardial infraction patients than the controls, which was attributed to the increased urinary loss of this element [101]. The possible protective role of Cr against CVD was indicated as in a case-control study conducted involving CVD patients, where the toenail Cr levels were higher for the control participants [102].

### 2.2. Cases with Different Smoking Habits and Residence Areas

Data obtained from cases’ serum was further classified depending on sex, smoking habit (current smokers, ex-smokers, and non-smokers) and the area of residence (urban, industrial, and rural).

#### 2.2.1. Sex

**Figure 5:**
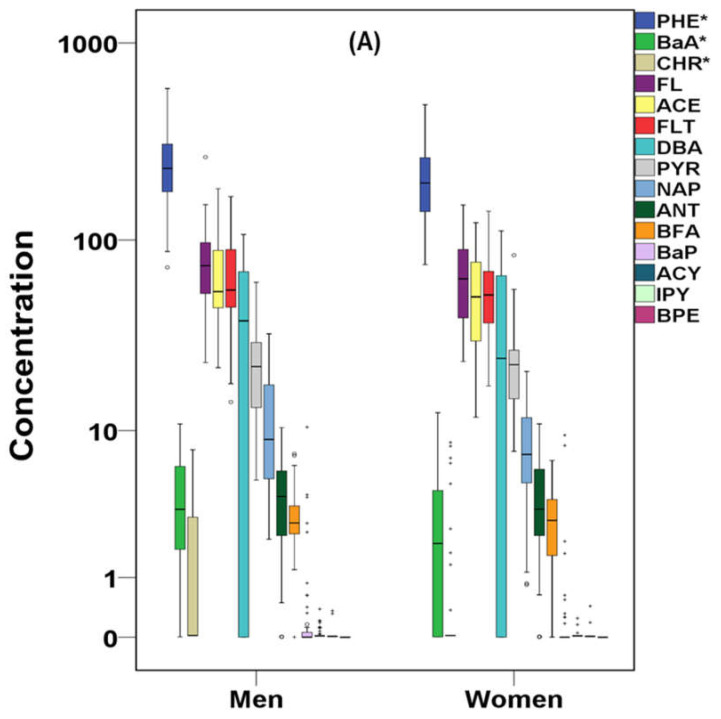

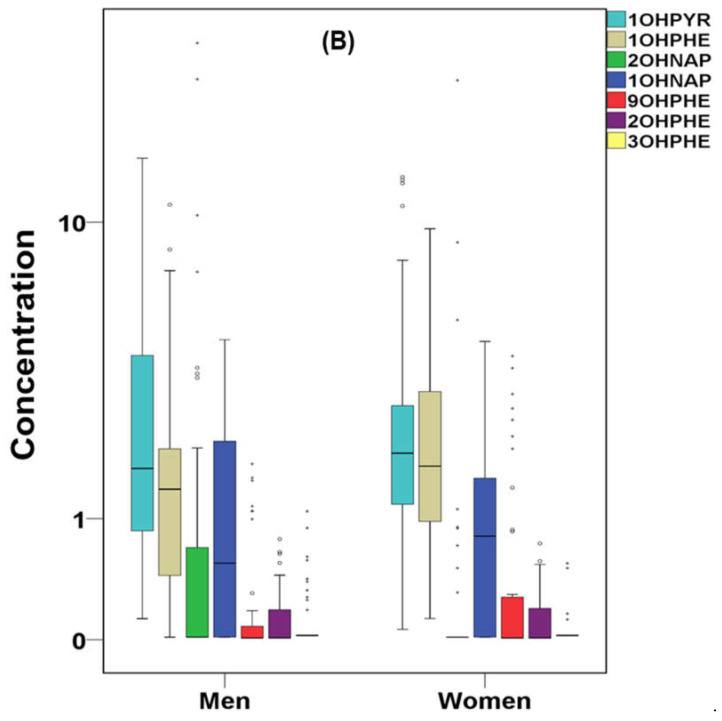

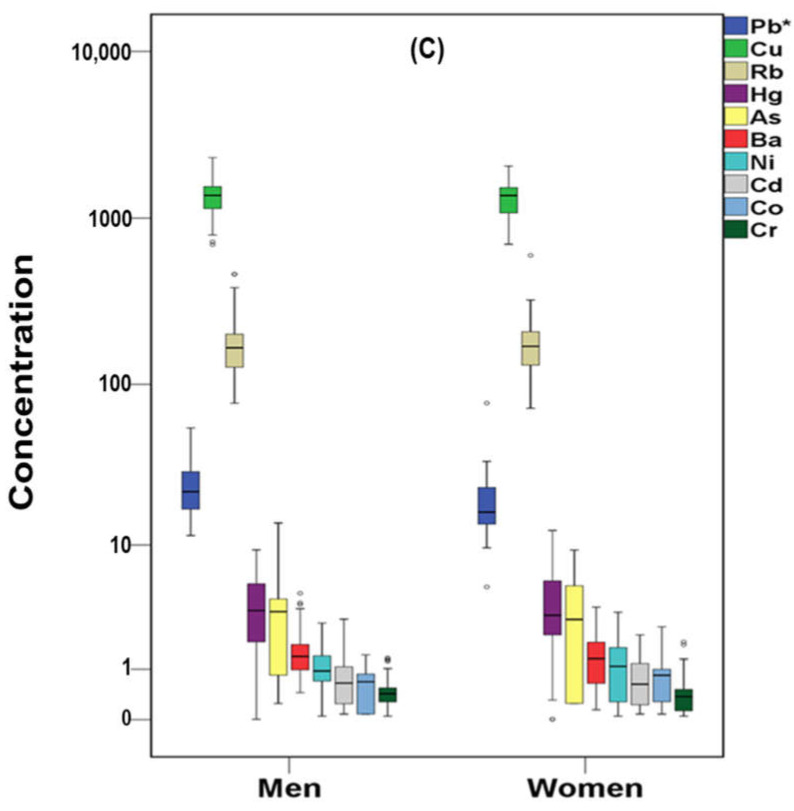
PAHs (**A**), OHPAHs (**B**), and trace elements’ (**C**) variation according to the gender of cases in μg L^−1^ (^+^ outliers and ^o^ values above 3rd quartile, * *p* < 0.05) in logarithmic scale (PHE:phenanthrene, BaA:benzo[a]anthracene, CHR:chrysene, FL:fluorene, ACE:acenaphthene, FLT:fluoranthene, DBA:dibenzo[a,h]anthracene, PYR:pyrene, NAP:naphthalene, ANT:anthracene, BFA:benzo[b,k]fluoranthenes, BaP:benzo[a]pyrene, ACY:acenaphthylene, IPY:indeno[1,2,3 cd]pyrene, BPE:benzo[ghi]perylene, 1OHPYR:1-hydroxypyrene, 1OHPHE:1-hydroxyphenanthrene, 2OHNAP:2-naphthol, 1OHNAP:1-naphthol, 9OHPHE:9-hydroxyphenanthrene, 2OHPHE:2-hydroxyphenanthrene, 3OHPHE:3-hydroxyphenanthrene).

Gender-based variation is a common classification of the data in case-control and cohort studies [103]. Most of the analytes presented higher concentrations in men (Figure 5), although only a few differences were significant (*p* < 0.05). In particular, regarding PAHs, PHE, BaA, and CHR presented significantly (*p* < 0.05) higher concentrations. OHPAHs, on the contrary, did not present any significant gender-based variation, although 1OHPHE was clearly higher in men’s serum (*p* = 0.054). Among trace elements, only Pb presented statistically higher concentrations in men (*p* < 0.05), which is in agreement with a previous study involving Greek citizens from Athens Metropolitan Area [104]. Various population studies have presented higher (*p* < 0.05) concentrations in males’ blood or serum. Particularly, in Korean men, blood Hg and Pb were found at significantly higher levels [105]. In Brazil, Cu and Pb were significantly correlated with gender and age [106]. In France, mean levels of blood Pb and Zn were found significantly higher in men, whereas Co and Cr were found significantly higher in women [107]. In the serum of Chinese students (17–20 years old), Pb was significantly higher in men [79]. A possible reason for the higher observed Pb in men’s samples could be the higher male hematocrit levels, as lead tends to bind to erythrocytes [108].

#### 2.2.2. Smoking Habit

**Figure 6:**
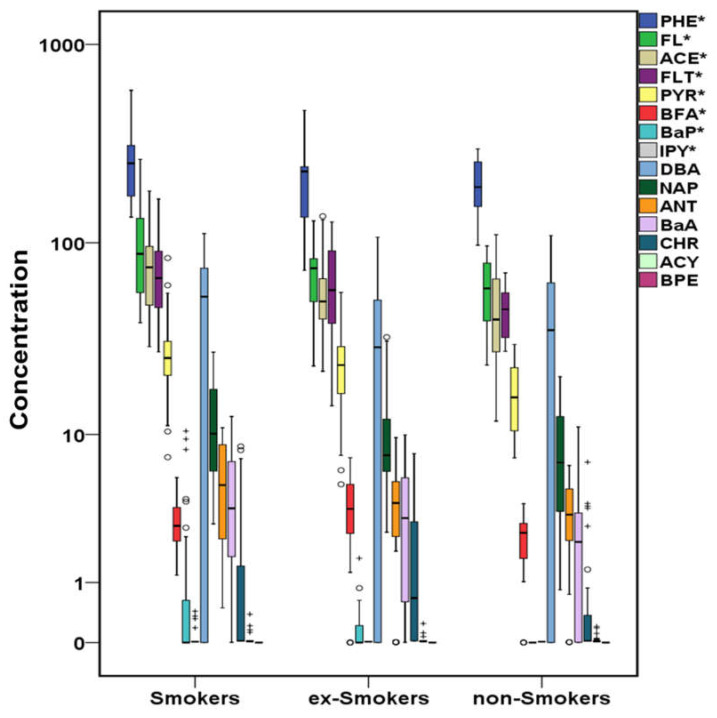
Variation of PAHs (μg L^−1^) in cases’ serum regarding the smoking habit (^+^ outliers, ^o^ values above 3rd quartile, and * *p*-value <0.05) in logarithmic scale (PHE:phenanthrene, FL:fluorene, ACE:acenaphthene, FLT:fluoranthene, PYR:pyrene, BFA:benzo[b,k]fluoranthenes, BaP:benzo[a]pyrene, IPY:indeno[1,2,3 cd]pyrene, DBA:dibenzo[a,h]anthracene, NAP:naphthalene, ANT:anthracene, BaA:benzo[a]anthracene, CHR:chrysene, ACY:acenaphthylene, BPE:benzo[ghi]perylene).

**Figure 7:**
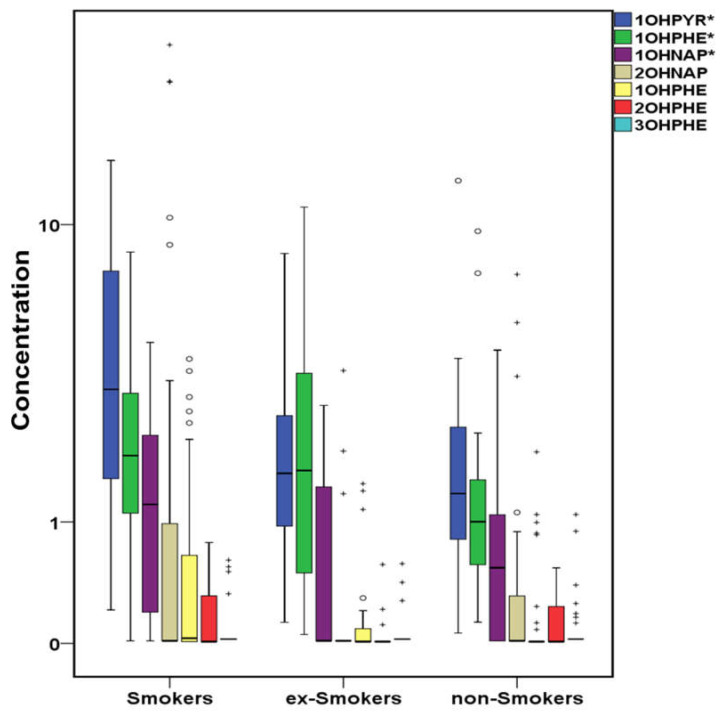
Variation of OHPAHs (μg L^−1^) in cases’ serum regarding the smoking habit (^+^ outliers, ^o^ values above 3rd quartile, and * *p*-value < 0.05) in logarithmic scale (1OHPYR:1-hydroxypyrene, 1OHPHE:1-hydroxyphenanthrene, 1OHNAP:1-naphthol, 2OHNAP:2-naphthol, 9OHPHE:9-hydroxyphenanthrene, 2OHPHE:2-hydroxyphenanthrene, 3OHPHE:3-hydroxyphenanthrene).

**Figure 8:**
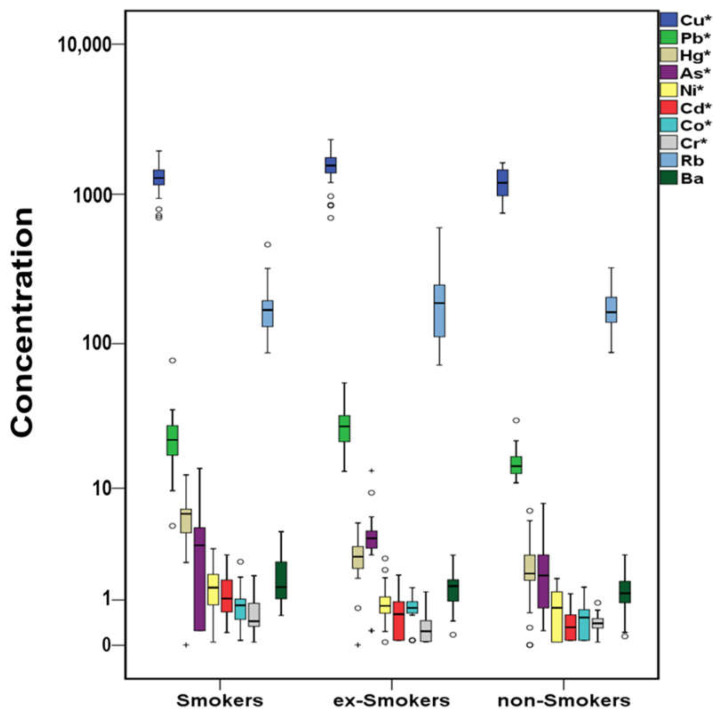
Variation of trace elements (μg L ^−1^) in cases’ serum regarding the smoking habit (^+^ outliers, ^o^ values above 3rd quartile and * *p*-value <0.05) in logarithmic scale.

Cigarette smoking is the major preventable cause of human death in the Western world being responsible for approximately 5 million annual premature deaths globally [109]. It is also a critical factor for CVD development and the second main cause of CVD mortality after high blood pressure [110]. Current and recent smokers are more vulnerable to smoking-related CVD risks than those who have quit smoking for a long time and non-smokers [111], with current or past smoking increasing the heart failure risk [112]. In Figure 6, Figure 7 and Figure 8, cases’ samples are classified in terms of the smoking habit. Among PAHs, PHE, FL, ACE, FLT, PYR, IPY (in a decreasing concentration sequence), and ΣPAHs (not shown in the figure) presented statistically higher concentrations in smokers’ serum followed by ex-smokers, while BFA and BaP were measured at higher levels in ex-smokers’ samples followed by smokers (Figure 6). HMW PAHs such as CHR, BaA, BaP and BPE were significantly higher in the serum of smoker leukemia patients [54]. In other studies, ACE and BaP were significantly higher in smokers’ samples, although the number of smokers in that study was limited [70]. Additionally, PAHs derivatives such as 1OHPYR, 1OHPHE, 1OHNAP, and ΣOHPAHs (not shown in the figure) were significantly higher in smokers (Figure 7). Although data about serum OHPAHs are limited, in smokers’ urine, higher levels of NAP, FL, PHE, and PYR metabolites have been frequently found compared to non-smokers’ urine [113,114].

Of trace elements, Hg, Ni, Cd, Co, and Cr were significantly higher in smokers’ serum (Figure 8) followed by ex-smokers, while Cu, As, and Pb were found statistically higher in ex-smokers’ serum followed by smokers. Ba and Rb did not present any noticeable trends. It is well known that tobacco and cigarette smoke contain plenty of trace elements including aluminum (Al), As, Ba, Beryllium (Be), Cd, Cr, Co, Cu, Fe, Hg, manganese (Mn), Ni, Pb, etc. [115]. Blood Pb and Hg levels were elevated compared to those of non-smokers [105]. A noteworthy increase of blood Cd levels have been related with smoking [114,116]. Particularly, in Greece, the blood Cd concentration of smokers has been found to be almost three times higher compared to non-smokers [104]. Nevertheless, significantly higher levels of blood Cd and Pb but lower blood Co and Hg have been also reported for smokers compared to non-smokers [107].

#### 2.2.3. Area of Residence

**Figure 9:**
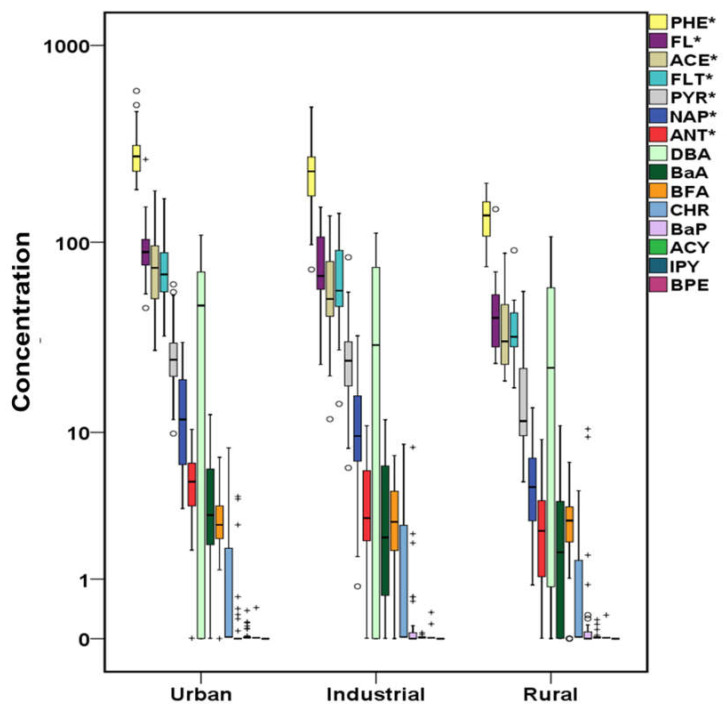
PAHs variation according to the area of residence of cases in μg L^−1^ (^+^ outliers and ^o^ values above 3rd quartile and * *p*-value <0.05) in logarithmic scale (PHE:phenanthrene, FL:fluorene, ACE:acenaphthene, FLT:fluoranthene, PYR:pyrene, NAP:naphthalene, ANT:anthracene, DBA:dibenzo[a,h]anthracene, BaA:benzo[a]anthracene, BFA:benzo[b,k]fluoranthenes, CHR:chrysene, BaP:benzo[a]pyrene, ACY:acenaphthylene, IPY:indeno[1,2,3 cd]pyrene, BPE:benzo[ghi]perylene).

**Figure 10:**
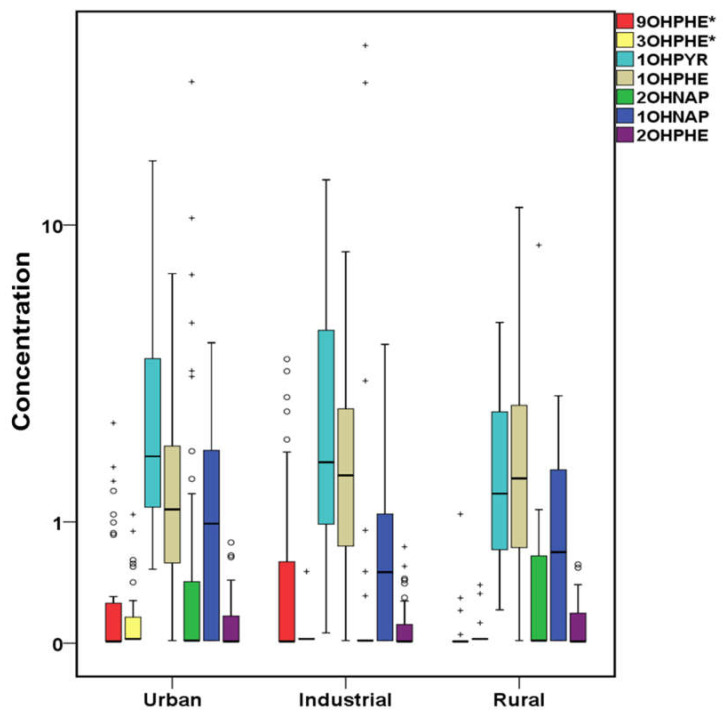
OHPAHs variation according to the area of residence of cases in μg L^−1^ (^+^ outliers and ^o^ values above 3rd quartile and * *p*-value <0.05) in logarithmic scale (9OHPHE:9-hydroxyphenanthrene, 3OHPHE:3-hydroxyphenanthrene, 1OHPYR:1-hydroxypyrene, 1OHPHE:1-hydroxyphenanthrene, 2OHNAP:2-naphthol, 1OHNAP:1-naphthol, 2OHPHE:2-hydroxyphenanthrene).

**Figure 11:**
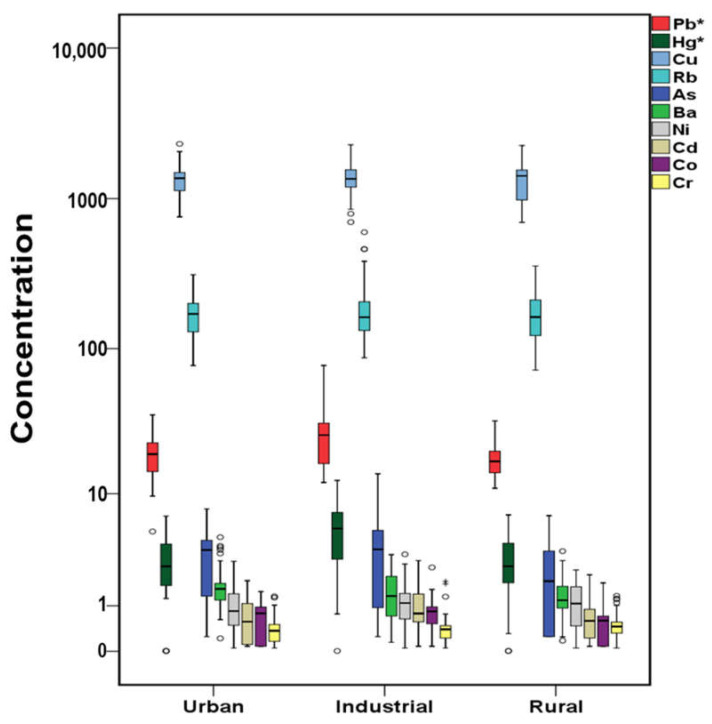
Trace elements variation according to the area of residence of cases in μg L^−1^ (^+^ outliers and ^o^ values above 3rd quartile and * *p*-value <0.05) in logarithmic scale.

In a recent study in Greece, air pollution was associated with mortality in both urban and rural areas [117]. After the economic crisis, the air quality of Athens and Thessaloniki, the two major Greek cities where almost the 50% of the population lives, was significantly degraded, especially during the winter months, due to biomass burning for domestic heating [118,119,120,121]. During crisis, there is evidence of increased number of CVD prevalence, although the atmospheric impact on CV system was not taken into consideration [122]. Significant amounts of PM, particle bound trace elements, and gas/particle phase PAHs have been measured in the Greater Athens Area [7,123], with a recent study highlighting combustion processes emissions as the crucial contributor to the PM_2.5_ and PM_1_ mass [124]. In Figure 9, Figure 10 and Figure 11, cases’ data are classified among the area of residence. As shown in Figure 9 and Figure 10, significantly higher (*p* < 0.05) concentrations of PHE, FL, ACE, FLT, PYR, NAP, and ANT as well as 3OHPHE from the OHPAHs group have been measured in the serum of urban site residents, followed by those of industrial sites, while in the serum of rural sites residents, the lowest PAHs concentrations were observed, except for ACY, revealing an important contribution of atmospheric PAHs yields to the serum concentrations. PAHs do not enter the human body only via inhalation, with food intake being also among the major sources [125]. As a result, the relative contribution of airborne PAHs, compared to other sources, regarding the CVD development, relies on location, dietary habits etc., although most of the PAHs that are taken up by the gastro-intestinal tract are subjected to first-path metabolism and eliminated in the liver [28]. On the other hand, inhaled BaP is absorbed in the alveolar region, enters the circulation, and reaches the heart and vasculature unmetabolized [28,126]. In our previous study, area of residence appeared to be an important contributor to the enhanced levels of ACY, BaP, IPY, and BPE in leukemia patients’ serum [54].

By contrast, the toxic Pb and Hg were found at statistically higher levels in the serum of industrial site residents (Figure 11), with the lowest concentrations of most metals observed in the samples of rural site residents, further implying the possible contribution of the local air quality on serum’s concentrations. In other studies, Pb blood levels were elevated in urban areas citizens compared to those of industrial and rural, while Hg was higher in the industrial citizens’ samples [127]. Significant associations between airborne trace elements and the corresponding serum levels were also found in a study conducted in China, with those who lived in urban sites, under elevated Pb and Cd emissions, presenting increased levels in their serum [79].

### 2.3. Principal Component Analysis (PCA)

PCA was carried out in order to assess any potential data patterns and to evaluate the possible relationships among the analytes that may occur. PCA is widely used in environmental studies [7,128,129]; nevertheless, applying PCA in biological samples may provide useful information for discussion and further research [54,130]. In this work, PCA was applied in cases and controls datasets separately (Table 1). Three components were chosen for PCA due to the fact that components after the fourth component explained variance less than 6.5%, and they could not have an adequate meaning of the results. Particularly, three components explained 37.0 and 35.8% of the variance in the cases and controls datasets, respectively. Of cases’ data, Factor 1 (18.2%) was tightly loaded with most of the LMW PAHs (except for ACY) FLT, PYR, 2OHPHE, 3OHPHRE, and OH1PYR, while the HMW PAHs were mostly clustered in Factor 3 (8.3%). The distribution of PAHs in the two factors could be explained by the different mechanisms of the compounds. For example, a recent study suggested that HMW PAHs including BFA, IPY, BaP, DBA, and others are crucial activators of AhR-mediated enzyme expressions [131]. On the contrary, LMW PAHs such as PHE are weak AhR agonists [132], having different unique cardiotoxicity mechanisms [26]. Factor 2 (10.3%) is loaded mostly by trace elements, with Cd, Co, Hg, and Pb presenting strong correlation. Regarding the metal toxicity, there are general mechanisms applied to all toxic metals, although some individual metals present additional unique mechanisms [133]. For instance, As, Cd, Hg, and Pb generate multiple reactive oxygen species, promoting oxidative stress [92,133]. In addition, Cd and Pb compete with the essential Zn, as they have similar physicochemical properties, for the binding sites of enzyme proteins [134]. As a result, the strong relation among some specific metals can be indicative of a similar mechanism, although the metal-induced cardiotoxicity mechanisms vary widely, and there is still a great matter of research [77]. Interestingly, the PCA that refers to the controls’ samples is completely different, with mixed factors and less significant correlations, supporting the hypothesis that PAHs and trace elements constitute a notable factor of heart failure development.

The latter hypothesis was further evaluated by applying logistic regression model, which is a tool generally used for the analysis of the relationship between individual risk/protective factors and outcomes [135]. Briefly, a new PCA was performed in the whole dataset including both cases’ and controls’ samples (Table S4). The regression-based factor scores of each sample for each of the three principal components (not shown), derived from the PCA, were saved as variables. Then, they were used as the input independent values and the clinical outcome (control or case occurrence) as the dependent variable for the regression analysis [136]. The Hosmer and Lemeshow Test (goodness-of-fit) indicated that the model is a satisfactory fit to the data (Table S5). Our results (Table 2) showed that every factor was statistically significant (*p* < 0.05), implying the plausible role of the studied compounds to heart failure development. It is noteworthy to mention that the strongest correlation was observed for Factor 2 (highest exp(B) value), which was mostly related with trace elements, supporting the outcome of the meta-analysis of Chowdhury et al. 2018, who highlighted the positive and approximately linear association of As, Pb, and Cd exposure with the risk of CVDs [137].

PCA was also performed according to the classification regarding the residence area as discussed in 2.2.3. As shown in Table S6, when the analysis refers to industrial sites, Factor 1 was dominated by the majority of PAHs. In addition, regarding the urban sites, the first two factors were loaded with LMW PAHs and their hydroxylated derivatives. Such trends were not observed in the analysis referring to rural areas, suggesting that air quality degradation could possibly be an important contributor to serum PAHs levels [54].

### 2.4. Strengths and Weaknesses

The present paper reports preliminary results on the relation of specific organic compounds, their metabolites, and trace elements with heart failure, using the approach of their direct measurement in human serum of cases and controls. To our knowledge, these kinds of results are reported for the first time. The findings of this study could provide useful insights about the abundance of PAHs and trace elements levels in patients serum, which are quite limited. In addition, through statistical analysis, atmospheric degradation and smoking appear to be significant contributors to the elevated serum levels of some pollutants and thus possibly enhance the development of heart failure.

However, this study does not provide evidence that PAHs, OHPAHs, and trace elements in serum are biomarkers for heart failure and it is clearly not an epidemiological study. The findings from this study should be interpreted with caution, as the population size, unknown factors related with CVDs (blood pressure, total cholesterol, or high-density lipoprotein cholesterol) and the lack of information for the dietary habits of the participants could set limits to the outcome. The findings of this study are based on a small dataset; thus, the statistics could be limited by confounding factors. Further studies are warranted regarding patients with specific disease such as CAD, or specific target groups, to estimate the possible CV risk from the elevated levels of PAHs and/or trace elements. The use of other biological matrices including whole blood, urine, and/or hair together with serum should be included in a future study.

## 3. Materials and Methods

### 3.1. Study Population

Ninety-six heart failure patients (cases) were recruited in General Hospital of Athens “Laiko” and participated on a voluntary basis. We defined incident heart failure diagnosis as the first record of heart failure in hospital admission records from any diagnostic position. Thirty-five healthy subjects (controls) were recruited as a reference. Inclusion criteria were the following: (1) the number of male and female participants should not differ significantly for both cases and controls (*p* > 0.05); (2) all participants should live in different residence areas, all over Greece, with different air quality levels; (3) the number of participants who were current smokers, ex-smokers, and non-smokers, should not differ significantly for both cases and controls (*p* > 0.05); (4) age of the participants more than 18 years; (5) long-term residence in the same area criterion was established (>5 consecutive years in the same area). The exclusion criteria were as follows: (1) all participants should not take any mineral supplement; (2) healthy subjects should not suffer for any other known disease (e.g., infectious disease). Each participant was informed in detail about the aims of the study and signed a written protocol. The study was approved by the Scientific Committee of “Laiko” Hospital, 1499/16/11/2017, according to the Helsinki Declaration. A detailed questionnaire was filled out by all participants and personal information was collected, such as gender, age, area of residence (urban, industrial, rural), and current smoking status (current smoker, ex-smoker, nonsmoker). Main socio-demographic characteristics of both controls and cases are shown in Table 3. Binomial test, for two variables, and Chi square test, for >2 variables, were carried out, respectively, with the hypothesis of equal probabilities. A value of *p* < 0.05 (95% confidence level) was considered to indicate a significant difference and thus retain the null hypothesis. The study focused on the different environmental factors, such as smoking and air quality of the residence area, which could possibly affect the analytes’ serum concentration.

### 3.2. Blood Sampling and Pretreatment

Blood samples were collected by qualified personnel at the General Hospital of Athens “Laiko”, during 2018. A total of 131 samples were obtained, from which 96 refer to cases (heart failure patients) and 35 refer to controls (healthy donors). From each participant, approximately 5 mL of blood were collected. The samples were centrifuged within 30 min for 10 min at 4500 rpm in order to separate serum from the cellular components. They were stored at −67 °C, until their transfer to our laboratory for further analysis (Laboratory of Analytical Chemistry, NKUA). Storage tubes were tested for any contamination by recovery tests using same solvents as in the procedure.

### 3.3. PAHs and OHPAHs Analysis and Quality Control

The extraction, clean up, and derivatization procedure has been described in detail in previous work [60]. Briefly, after the addition of internal standards, extraction was performed in an ultrasonic bath, followed by a pre-concentration step with a rotary evaporator. The concentrated sample was cleaned up using glass column chromatography. The eluted fraction containing PAHs was adjusted at 0.5 mL using a gentle steam of nitrogen, while the fraction containing OHPAHs was gently evaporated until dryness followed by the addition of the derivatization reagents, i.e., 250 μL of *N,O*-bis(trimethylsilyl)trifluoroacetamide (BSTFA) with 1% trimethylchlorosilane (TMCS) and 50 μL of anhydrous pyridine. The reaction took place in an oven at 70 °C for 3 h. A gas chromatography/mass spectrometry system (GC/MS) (6890N/5975B, Agilent Technologies, USA) was employed for the determination of both fractions. The GC instrument was equipped with a split/splitless injector and an HP-5ms (5%-(phenyl)-methylpolysiloxane) (Agilent J&W GC Columns, Agilent Technologies, Santa Clara, CA, USA) capillary column. High-purity helium was used as carrier gas with a velocity of 1.5 mL min^−1^. Pulsed split-less mode was used for the injection and the injector’s temperature was set at 280 °C. For PAHs and OHPAHs analysis, the GC oven temperature program was 65 °C (hold for 1 min) to 320 °C at 15 °C/min with final isothermal hold for 3 min. In both cases, inlet and MS source temperatures were 280 and 230 °C, respectively. The selected ion monitoring (SIM) mode was used for the quantification of the analytes.

The PAH-determination procedure was validated using the Polynuclear Aromatic Hydrocarbons Mix (Supelco, Darmstadt, GER), which is a standard solution of the compounds studied including NAP, ACY, ACE, FL, PHE, ANTH, FTL, PYR, CHR, BaA, BFA, BaP, IPY, DBA, and BPE. In the same way, a OHPAHs mix was prepared including the following compounds: 1OHNAP, 2OHNAP, 1OHPHE, 2OHPHE, 3OHPHE, 9OHPHE, and 1OHPYR. Recovery rates and selectivity were evaluated using spiked blood serum. Blank samples, i.e., mixtures of controls’ serum obtained by non-smoking residents of low polluted areas (mainly from Greek islands), were pretreated in the same way and analyzed in order to examine the potential background effect. Recovery rates, LoD, and limits of quantification (LoQ) are shown in Table S7. In general, recoveries ranged from 72.5% (1OHNAP) to 136% (ACY) and LoD varied from 0.001 (BaP) to 0.11 (NAP) μg L^–1^.

### 3.4. Trace Elements’ Analysis and Quality Control

All plastic materials that came into contact with the serum samples were previously washed thoroughly, soaked in dilute nitric acid (HNO_3_) (Merck, Darmstadt, Germany), and rinsed with ultrapure water of 18.2 MΩ cm (Millipore, Bedford, MA, USA). The followed pretreatment procedures have been described elsewhere [138,139]. Briefly, 0.5 mL of serum were wet digested using a mixture of HNO_3_ (suprapur 65%) and hydrogen peroxide (H_2_O_2_) (suprapur 30%) (Merck). The samples were analyzed with inductively coupled plasma mass spectrometry (ICP-MS) by a Thermo Scientific ICAP Qc (Waltham, MA, USA). Measurements were carried out in a single collision cell mode, with kinetic energy discrimination (KED) using pure He. Matrix induced signal suppressions and instrumental drift were corrected by internal standardization (^45^Sc, ^103^Rh).

In each batch of 10 samples, at least one laboratory blank was analyzed. In case trace element concentrations in the reagent blank were detectable, the procedure for the whole batch was repeated. In order to verify the accuracy and precision of the method, the certified reference materials (CRM) “Plasma Control lyophilized, Levels I and II” (RECIPE Chemicals + Instruments GmbH, Munich, Germany) were used. The recoveries for As, Cd, Co, Cr, Cu, Ni, and Pb ranged from 95.1 to 105% (certified values for Ba, Hg and Rb are not included in the specific certified reference materials). The USEPA method [140] was applied for the calculation of the LoD and LoQ. LOD ranged from 0.10 μg L^–1^ (Cr) to 0.7 μg L^–1^ (Ba).

### 3.5. Statistical Analysis

The SPSS software package (IBM SPSS statistics version 24) was employed for statistical analysis purposes. SPSS software is a common tool for statistical analysis of data from environmental including air [7,141,142], water [143], and soil [144] samples or biological [60,145] samples.

Hypothesis tests for population proportion were carried out using binomial test (2 variables) and chi-square test (3 or more variables). *p* values > 0.05 indicate non–significant difference. The normal distribution of the data was assessed using the Shapiro–Wilk and Kolmogorov–Smirnov tests, with a value of *p* > 0.05 indicating normal distribution. As no variable of the dataset was normally distributed, the possible statistical differences between two or more independent variables were investigated using the Mann–Whitney and Kruskal–Wallis tests, respectively, with the value of *p* < 0.05 indicating statistically significant difference. Principal Component Analysis (PCA) was used for the investigation of any possible associations of the examined parameters. PCA is a widespread multivariate statistical technique used in environmental sciences [54,141,142,145]. The application of PCA transforms the original set of variables into a smaller one of linear combinations accounting for the most of the variance of the former set. It makes the complex system more accessible, while at the same time it withholds the primary information. Varimax rotation is generally used for factor grouping in most PCA applications. The exported principal components include variables with common characteristics, which are attributed as a common source or chemical interaction. [146,147]. It should be noted that the variables used in this study were standardized before applying PCA.

Further statistical analysis includes the application of logistic regression after PCA. In logistic regression, the relationship between a binary dependent variable, for example, the occurrence of a phenomenon or not, with independent variables, which affect that phenomenon, is assessed, as generally used in medical and epidemiological studies. Although logistic regression has many similarities with linear regression, the estimation of variables’ coefficients is performed by the maximum likelihood technique [148]. Under this prism, combination of logistic regression with PCA could reveal the probability of each factor to be associated with the occurrence of heart failure.

## 4. Conclusions

Major environmental pollutants have been measured in the serum of heart failure patients and have been compared with control samples. The statistical higher concentrations of the majority of PAHs, especially the low molecular weight, and trace elements indicate a potential link with heart failure. Smoking habit and atmospheric degradation of urban and industrial sites appeared to further elevate the analytes’ serum concentrations. Possible common mechanisms related to heart failure are revealed from principal component analysis followed by logistic regression model, suggested some of the analytes as possibly significant contributors to heart failure incidence. As a future aspect, a fully designed study with a more specific patient group and a wider dataset of biochemical parameters will provide additional information for the further evaluation of the role environmental compounds to CVDs.

## Figures and Tables

**Table 1:** Varimax rotated PCA for PAHs, OHPAHs, and trace elements for cases’ and controls’ samples. (Loadings > 0.600 appeared in bold).

	Cases			Controls		
Variance %	18.2	10.5	8.3	15.0	12.0	8.8
	1	2	3	1	2	3
PHE	**0.873**	−0.100	0.137	0.074	**0.631**	−0.481
FLT	**0.866**	0.016	0.159	0.215	0.187	−0.238
ACE	**0.804**	0.211	0.059	0.089	0.295	−0.394
NAP	**0.764**	−0.063	0.171	0.505	−0.302	−0.112
1OHPYR	**0.741**	0.362	−0.334	−0.126	**0.704**	0.247
FL	**0.735**	0.090	0.237	0.022	0.118	−0.020
PYR	**0.694**	0.177	0.289	−0.240	**0.639**	0.164
2OHPHE	**0.692**	−0.111	−0.129	0.553	0.365	−0.213
ANT	**0.676**	0.101	0.209	0.490	0.219	0.022
3OHPHE	**0.634**	−0.067	−0.252	0.016	0.268	0.589
Cd	0.139	**0.704**	0.122	−0.113	0.196	−0.365
Pb	0.138	**0.631**	0.118	**0.613**	0.089	0.203
Co	0.011	**0.624**	0.091	−0.325	−0.440	−0.016
Hg	0.015	**0.601**	0.118	0.110	−0.041	−0.002
CHR	0.061	−0.051	**0.728**	**0.604**	0.012	−0.042
BaA	0.313	−0.009	**0.614**	0.452	0.007	0.173
DBA	−0.118	−0.172	**0.610**	−0.012	**0.746**	0.146
BFA	0.189	0.073	**0.606**	0.482	0.514	0.083
ACY	0.290	0.027	0.033	0.536	−0.192	0.214
BaP	−0.034	0.360	−0.006	**0.901**	−0.024	0.057
IPY	−0.038	0.155	0.324	**0.803**	0.231	−0.176
1OHNAP	0.023	0.507	−0.038	0.005	0.261	**0.644**
2OHNAP	0.200	0.420	−0.142	−0.006	−0.094	0.477
1OHPHE	−0.098	0.473	−0.255	0.314	0.291	0.225
9OHPHE	0.368	0.264	−0.221	−0.003	−0.258	0.057
As	0.014	0.364	0.303	0.229	0.239	0.099
Ba	0.419	0.215	−0.174	0.386	0.028	−0.244
Cr	0.063	0.432	−0.188	0.166	0.252	**0.776**
Cu	0.031	0.303	−0.150	0.464	−0.487	0.256
Ni	−0.026	0.522	0.244	0.338	−0.040	−0.233
Rb	−0.197	0.048	−0.217	−0.005	0.457	−0.030

**Table 2:** The result of the logistic regression analysis.

	**B**	**S.E.**	**Wald**	**Sig.**	**Exp(B)**	**95% C.I. for EXP(B)**	
						**Lower**	**Upper**
**Regression Scores Factor 1**	3.375	1.518	4.945	0.026	29.214	1.492	571.879
**Regression Scores Factor 2**	10.534	3.868	7.418	0.006	37,561.068	19.167	73,6079,32.642
**Regression Scores Factor 3**	4.592	1650	7.747	0.005	98.668	3.890	2502.895

**Table 3:** Demographic characteristics and smoking status for controls and cases.

	Cases (*n* = 96)			Controls (*n* = 35)		
	*N*	(%)	*p* Value	*N*	(%)	*p* Value
**Sex**						
Men	52	54.2	0.475	18	51.4	0.999
Women	44	45.8		17	48.6	
**Residence Area**						
Industrial	31	32.3	0.380	15	42.9	0.499
Urban	38	39.6		20	57.1	
Rural	27	28.1		-	-	
**Age (Years)**						
40–49	12	12.5	0.004	15	42.8	0.449
50–59	26	27.1		11	31.4	
60–69	27	28.1		9	25.7	
70–79	23	24.0		-	-	
80–89	8	8.3		-	-	
**Smoking Habit**						
Ever	36	37.5	0.519	11	31.4	0.892
Ex	27	28.1		11	31.4	
Never	33	34.3		13	37.1	

## Data Availability

The data may be available from the corresponding author on.

## Supplementary Materials

The following are available online, Table S1: Detection frequencies, median, mean and ranges of PAHs concentrations (μg L^−1^) in cases’ and controls’ samples, Table S2: Detection frequencies, median, mean and ranges of OHPAHs concentrations (μg L^−1^) in cases’ and controls’ samples, Table S3: Detection frequencies, median, mean and ranges of trace elements’ concentrations (μg L^−1^) in cases’ and controls’ samples, Table S4: Varimax rotated PCA for PAHs, OHPAHs and trace elements for overall dataset used for logistic regression model, Table S5: Hosmer and Lemeshow Test, Table S6: Varimax rotated PCA for PAHs, OHPAHs and trace elements for cases’ samples classified in terms of the residence area. (Loadings > 0.600 appeared in bold), Table S7: Analytical method recovery rates, LoD and LoQ for the determination of PAHs and OH-PAHs in human serum.

## Sample Availability

Samples of the compounds are not available from the authors.
