# Supplementary material for: Heart Failure and PAHs, OHPAHs, and Trace Elements Levels in Human Serum: Results from a Preliminary Pilot Study in Greek Population and the Possible Impact of Air Pollution
Source: Molecules. 2021 May 27;26(11):3207. doi: 10.3390/molecules26113207 (PMC8199329; doi:10.3390/molecules26113207)
Supplement: Supplementary file 1 [file molecules-26-03207-s001.zip › molecules-1211455-supplementary.pdf]

Supplementary material of "**Heart failure and PAHs, OHPAHs and trace elements levels in human serum: results from a preliminary pilot study in Greek population and the possible impact of different air pollution**"

Eirini Chrysochou[1], Panagiotis Georgios Kanellopoulos[1], Konstantinos Koukoulakis[1], Aikaterini Sakellari[2], Sotirios Karavoltsos[2], Minas Minaidis[3] and Evangelos Bakeas[1]*

[1]Laboratory of Analytical Chemistry, Department of Chemistry, National and Kapodistrian University of Athens, 6 Zografos, Panepistimiopolis, 15784 Athens, Greece

[2]Laboratory of Environmental Chemistry, Department of Chemistry, National and Kapodistrian University of 8 Athens, Zografos, Panepistimiopolis, 15784 Athens, Greece

[3]General Hospital "LAIKO", 11527, Athens, Greece

*corresponding author, Email: bakeas@chem.uoa.gr

Content

Table S1: Detection frequencies, median, mean and ranges of PAHs concentrations (μg L^{-1}) in cases' and controls' samples.

Table S2: Detection frequencies, median, mean and ranges of OHPAHs concentrations (μg L^{-1}) in cases' and controls' samples.

Table S3: Detection frequencies, median, mean and ranges of trace elements' concentrations (μg L^{-1}) in cases' and controls' samples.

Table S4: Varimax rotated PCA for PAHs, OHPAHs and trace elements for overall dataset used for logistic regression model.

Table S5: Hosmer and Lemeshow Test.

Table S6: Varimax rotated PCA for PAHs, OHPAHs and trace elements for cases' samples classified in terms of the residence area. (Loadings > 0.600 appeared in bold).

Table S7: Analytical method recovery rates, LoD and LoQ for the determination of PAHs and OH-PAHs in human serum.

Table S1. Detection frequencies, median, mean and ranges of PAHs concentrations (μg L^{-1}) in cases' and controls' samples.

	NAP	ACY	ACE	FL	PHE	ANT	FLT	PYR	CHR	BaA	BFA	BaP	IPY	DBA	BPE	ΣPAHs
**Cases (n=96)**																
Detection Frequency (%)	100	13.5	100	100	100	91.7	100	100	35.4	78.1	93.7	25.0	4.2	67.7	0	
Median	7.85	0.02	51.8	69.1	224	3.81	54.6	22.4	0.02	2.67	2.82	0.0004	0.01	35.8	ND	506
Mean	10.5	0.03	63.0	76.7	231	4.26	61.6	23.4	1.24	3.62	2.99	0.53	0.02	38.5	ND	517
Geometric mean	8.04	0.02	54.1	67.7	209	2.34	54.4	20.3	0.11	0.83	1.80	0.003	0.01	1.68	ND	473
Geometric SD	1.41	1.34	1.28	1.25	1.22	2.16	1.25	1.27	2.83	3.48	2.27	4.23	1.35	8.35	ND	1.21
Range	0.84-32.9	0.02-0.39	11.8-183	23.3-264	72.5-588	0.01-10.9	14.3-167	5.18-83.7	0.02-8.58	0.004-12.5	0.001-7.40	0.0004-10.4	0.01-0.44	0.001-111	ND	163-1160
**Controls (n=35)**																
Detection Frequency (%)	100	22.9	100	100	100	77.1	100	100	28.6	71.4	77.1	14.3	8.57	65.7	0	
Median	5.73	0.02	16.9	22.8	54.7	1.80	13.8	5.86	0.03	1.09	0.99	0.0004	0.01	9.44	ND	142
Mean	6.67	0.03	19.0	26.6	56.5	1.98	15.3	7.04	0.40	1.69	1.20	0.08	0.02	9.81	ND	146
Geometric mean	5.09	0.02	17.0	23.9	52.9	0.62	13.1	6.12	0.06	0.33	0.27	0.001	0.01	0.57	ND	142
Geometric SD	1.42	1.32	1.21	1.21	1.17	2.79	1.29	1.27	2.22	3.44	3.58	2.81	1.35	6.67	ND	1.12
Range	0.74-16.4	0.02-0.24	7.83-70.8	12.4-82.8	24.8-104	0.01-5.27	3.75-36.2	2.63-16.7	0.02-3.93	0.004-7.66	0.001-3.81	0.0004-1.16	0.01-0.26	0.001-28.3	ND	87.4-230
p-Value	0.007	0.266	0.000	0.000	0.000	0.000	0.000	0.000	0.190	0.003	0.000	0.163	0.357	0.000	-	0.000

Table S2. Detection frequencies, median, mean and ranges of OHPAHs concentrations (μg L^{-1}) in cases' and controls' samples.

	1OHNAP	2OHNAP	1OHPHE	2OHPHE	3OHPHE	9OHPHE	1OHPYR	ΣOHPAHs
	**Cases (n=96)**							
Detection Frequency (%)	65.6	25.0	96.9	31.2	14.6	36.5	100	
Median	0.71	0.02	1.48	0.01	0.03	0.01	1.87	5.34
Mean	1.02	1.40	2.10	0.12	0.09	0.37	3.13	8.24
Geometric mean	0.29	0.07	1.20	0.03	0.04	0.04	1.88	5.90
Geometric SD	2.45	2.56	1.73	2.03	1.50	2.41	1.58	1.41
Range	0.01-4.59	0.02-29.8	0.02-11.2	0.01-0.78	0.03-1.09	0.01-4.09	0.06-14.9	0.58-45.3
	**Controls (n=35)**							
Detection Frequency (%)	28.6	17.1	62.9	20.0	8.57	11.4	77.1	
Median	0.01	0.015	0.18	0.01	0.03	0.01	0.99	1.76
Mean	0.12	0.13	0.40	0.06	0.03	0.04	1.87	2.67
Geometric mean	0.02	0.03	0.13	0.02	0.03	0.01	0.63	1.57
Geometric SD	1.73	1.73	2.06	1.67	1.17	1.46	2.43	1.68
Range	0.01-1.54	0.01-1.76	0.01-1.95	0.01-1.09	0.03-0.25	0.01-0.80	0.01-8.67	0.11-10.7
p-Value	0.000	0.216	0.000	0.114	0.289	0.003	0.014	0.000

Table S3. Detection frequencies, median, mean and ranges of trace elements' concentrations (μg L^{-1}) in cases' and controls' samples.

	As	Ba	Cd	Co	Cr	Cu	Hg	Ni	Pb	Rb
					Cases (n=96)					
Detection Frequency (%)	75	100	80.2	73.9	100	100	93.7	86.5	100	100
Median	3.39	1.35	0.64	0.74	0.40	1369	3.33	0.98	19.8	166
Mean	3.25	1.53	0.76	0.69	0.47	1332	3.84	1.06	22.0	186
Geometric mean	1.87	1.26	0.49	0.46	0.32	1285	2.37	0.69	20.1	171
Geometric SD	1.74	1.35	1.60	1.60	1.52	1.12	1.96	1.67	1.20	1.19
Range	0.25-13.9	0.15-4.67	0.08-2.97	0.08-2.57	0.05-1.90	694-2308	0.01-12.4	0.05-3.37	5.18-77.0	71.6-597
**Controls (n=35**										
Detection Frequency (%)	60	85.7	57.1	60	94.2	100	71.4	68.6	82.9	100
Median	0.98	1.48	0.17	0.56	0.57	1037	0.68	0.23	6.44	178
Mean	1.26	1.44	0.22	0.69	0.70	1027	0.80	0.30	5.94	195
Geometric mean	0.77	1.24	0.16	0.36	0.51	1009	0.25	0.19	2.75	188
Geometric SD	1.57	1.31	1.39	1.76	1.48	1.08	2.54	1.59	2.30	1.13
Range	0.25-4.57	0.35-2.92	0.08-1.05	0.08-2.80	0.05-2.39	654-1583	0.01-3.57	0.05-0.94	0.05-13.8	111-353
p-Value	0.000	0.942	0.000	0.520	0.006	0.000	0.000	0.000	0.000	0.102

Table S4. Varimax rotated PCA for PAHs, OHPAHs and trace elements for overall dataset used for logistic regression model.

**Variance (%)**	**16.9**	**13.4**	**10.5**
	1	2	3
ACE	**.765**	.352	.220
PHE	**.748**	.256	.419
FLT	**.742**	.304	.401
NAP	**.690**	.016	.278
1OHPYR	**.686**	.302	-.299
FL	**.653**	.303	.399
PYR	**.643**	.362	.389
ANT	**.641**	.206	.311
3OHPHE	.544	-.042	-.157
2OHPHE	.456	-.113	.031
9OHPHE	.456	.246	-.168
Ba	.386	.135	-.117
ACY	.294	-.051	.001
Rb	-.207	.093	-.154
Cd	.182	**.710**	.103
Hg	.140	**.666**	.182
1OHPHE	-.003	**.641**	-.095
Ni	.080	**.604**	.251
1OHNAP	.127	.577	.016
Pb	.242	.543	.317
Cu	.091	.506	.065
As	-.001	.504	.369
2OHNAP	.216	.402	-.199
Co	-.061	.399	-.147
BaP	-.014	.371	-.035
CHR	.023	-.006	**.707**
DBA	-.054	.085	**.632**
BFA	.248	.250	**.601**
BaA	.288	.062	.585
Cr	.035	.094	-.370
IPY	.022	.012	.215

Table S5. Hosmer and Lemeshow Test.

Hosmer and Lemeshow Test			
**Step**	**Chi-square**	**df**	**Sig.**
1	.099	8	1.000

Table S6. Varimax rotated PCA for PAHs, OHPAHs and trace elements for cases' samples classified in terms of the residence area. (Loadings > 0.600 appeared in bold).

	**Urban**			**Industrial**			**Rural**		
Variance (%)	17.0	11.9	9.8	19.3	13.5	10.4	17.1	15.6	13.4
	1	2	3	1	2	3	1	2	3
NAP	.587	.337	-.241	**.759**	-.031	.149	.041	-.408	.079
ACY	.156	.537	.063	.439	-.129	-.162	-.128	.032	**.587**
ACE	.434	**.783**	-.063	**.696**	.388	.300	.412	.079	**.808**
FL	.374	.169	-.046	**.845**	.202	.116	**.933**	-.224	.058
PHE	**.804**	.285	-.151	**.857**	.081	-.229	.401	**-.619**	.164
ANT	.387	.312	-.003	**.792**	.147	.110	.554	.062	.273
FLT	**.709**	.388	-.075	**.880**	.132	-.154	**.849**	-.370	.110
PYR	**.820**	.218	.161	**.675**	.085	.306	.233	-.005	.243
CHR	.006	.000	.261	.399	**-.605**	.304	.023	-.225	**.805**
BaA	.272	.052	**.603**	.539	-.488	.206	.201	-.320	.537
BFA	.212	-.019	.336	**.664**	-.214	.101	-.069	-.009	**.627**
BaP	.305	-.075	.050	-.051	-.120	.412	**.678**	.337	-.179
IPY	-.068	-.016	.369	.033	-.178	**.587**	.085	.128	**.558**
DBA	-.453	-.135	-.121	.134	**-.669**	.466	.093	-.515	.340
OHNAP1	.274	-.251	.227	-.132	.366	**.550**	.366	**.809**	.126
OHNAP2	-.196	**.785**	-.013	.038	.454	.183	**.876**	.178	-.057
OHPHE1	-.163	**.611**	.109	-.130	.432	-.095	.028	**.631**	-.122
OHPHE2	**.719**	-.106	-.093	.157	.228	.269	-.038	-.518	-.041
OHPHE3	.419	.058	-.091	.271	.324	-.134	.186	-.155	.295
OHPHE9	**.598**	-.022	.021	.107	**.550**	.061	.006	.211	**.631**
OHPYR1	**.689**	.177	.096	.356	**.819**	.044	.507	.380	-.031
As	-.218	.085	**.600**	.215	-.099	-.219	.244	.566	.096
Ba	.269	.528	-.162	.183	**.562**	.195	.359	-.184	-.328
Cd	.331	.409	**.514**	.090	.216	.499	**.631**	.496	-.203
Co	-.195	.242	**.663**	-.190	.520	.073	.470	.296	.300
Cr	.154	.496	.045	-.035	.350	.494	.265	.582	.182
Cu	.047	.355	.323	-.187	.167	**-.610**	.103	.598	-.004
Hg	-.069	.000	.560	-.119	.480	**.620**	.504	.159	.079
Ni	.143	-.123	**.746**	.119	.334	.134	.363	.323	.185
Pb	.330	.488	.176	.147	-.007	-.168	.039	**.668**	.021
Rb	-.315	.049	-.143	-.206	-.029	-.426	.014	.135	-.588

Table S7: Analytical method recovery rates, LoD and LoQ for the determination of PAHs and OH-PAHs in human serum

NAME	Recovery rate				LOD ng mL^{-1}	LOQ ng mL^{-1}
	**25ng mL^{-1}**	**RSD%**	**50ng mL^{-1}**	**RSD%**		
**NaP**	110	10.2	82.6	30.3	0.11	0.32
**ACY**	136	17.7	132	8.09	0.03	0.09
**ACE**	96.5	5.94	128	9.42	0.03	0.01
**FL**	99.1	10.8	124	6.38	0.02	0.07
**PHE**	89.4	60.1	104	10.1	0.03	0.10
**ANT**	138	19.9	131	7.72	0.01	0.034
**FLT**	106	14.3	96	1.41	0.09	0.27
**PYR**	98.3	2.43	107	7.54	0.09	0.27
**CHR**	122	13.1	131	0.33	0.04	0.12
**BaA**	77.8	8.10	80.8	1.07	0.01	0.026
**BFA**	70.2	11.1	84.9	6.83	0.003	0.009
**BaP**	112	8.61	114	15.5	0.001	0.003
**IPY**	89.4	10.2	81.3	9.24	0.02	0.07
**DBA**	79.0	10.0	72.5	14.2	0.003	0.009
**BPE**	78.7	9.35	70.9	4.23	0.002	0.006
**1OHNAP**	72.5	10.9	77.7	16.7	0.03	0.09
**2OHNAP**	77.3	12.3	79.1	14.2	0.03	0.09
**1OHPHE**	82.1	9.9	83.2	12.2	0.03	0.09
**2OHPHE**	86.7	8.8	85.6	14.3	0.02	0.06
**3OHPHE**	88.0	10.4	89.4	12.2	0.05	0.15
**9OHPHE**	93.1	8.6	92.1	9.7	0.02	0.06
**1OHPYR**	91.8	10.3	94.4	10.9	0.03	0.09
